# A prospective randomized comparison of two skin closure techniques in acetabular fracture surgery

**DOI:** 10.1007/s10195-013-0282-7

**Published:** 2013-12-31

**Authors:** Christopher D. Mudd, John A. Boudreau, Berton R. Moed

**Affiliations:** 1 Metropolitian Orthopedics, Missouri Baptist Medical Center, 3009 Ballas Road, Suite 105 B, St. Louis, MO 63131 USA; 2 Department of Orthopaedic Surgery, Saint Louis University School of Medicine, 3635 Vista Avenue, 7th Floor Desloge Towers, St. Louis, MO 63110 USA

**Keywords:** Skin closure, Acetabular fracture, 2-Octyl cyanoacrylate

## Abstract

**Background:**

Recent publications have shown an infection rate of 5–7 % for acetabular fractures treated with the Kocher-Langenbeck (K-L) approach. Using metallic staples to close hip skin incisions has been considered the gold standard. The purpose of this study was to answer the following: (1) will closure of a K-L incision after acetabular fracture surgery with a running subcuticular monocryl suture, then sealing the wound with 2-octyl cyanoacrylate (OCA), result in a lower infection rate compared to metallic staple closure? (2) Do incisions closed with subcuticular monocryl and OCA exhibit decreased drainage? (3) Is there a cost difference between these two methods?

**Materials and methods:**

In a prospective clinical study, 103 patients with acetabular fractures treated using the K-L approach were randomized into two groups: skin closure with metallic staples (*n* = 52) versus subcuticular running monocryl suture sealed with OCA (*n* = 51).

**Results:**

Two postoperative deep infections (4 %) in the staples group required multiple debridements; no infections developed in the OCA group. However, there was no statistical difference between the groups, (*p* = 0.495). There was a statistically significant difference (*p* = 0.032) comparing days from surgery to a dry incision favoring OCA (4.2 versus 5.85 days). The patient charge was approximately $900 greater on average in the OCA group due to the increased time in the operating room required for the subcuticular closure.

**Conclusions:**

Closure with OCA and subcuticular monocryl showed no clinical disadvantages and appears to have a clinical advantage when compared to standard metallic staple skin closure in acetabular fracture surgery. However, additional patient costs may be incurred.

**Level of evidence:**

II.

## Introduction

Metallic skin staples have served as a primary method of superficial skin closure in surgery of the hip [1–3]. The use of 2-octyl cyanoacrylate (OCA; Dermabond, Ethicon, Newark, NJ, USA) has been shown to be a safe and effective alternative to metallic staple closure of both surgical and traumatic wounds [4–10]. Recent publications have shown decreased infection rates, reduced wound drainage, and improved cosmetic satisfaction when comparing OCA closure, with and without sutures, to skin staples in total hip arthroplasty [3, 11–14]. In addition, OCA has demonstrated bacteriostatic effects [15–17].

The Kocher-Langenbeck (K-L) approach [1] to the acetabulum requires an extensive deep dissection. In already traumatized soft tissues, the surgical wound is at risk for complications such as persistent drainage and deep infection. Deep infection of the acetabulum is a devastating complication with extensive destruction to the articular cartilage of the hip joint [18]. Recent publications have shown an infection rate of 5–7 % for acetabular fractures treated with a K-L approach [19–22]. We are unaware of any existing study comparing different wound closure techniques in acetabular fracture surgery. It was our hypothesis that the potential advantages of OCA demonstrated in the arthroplasty literature may be applicable to acetabular fracture surgery.

The purpose of this blinded randomized controlled trial was to answer the following questions: (1) will closure of a K-L incision after acetabular fracture surgery with a running subcuticular monocryl suture, then sealing the wound with OCA, result in a lower infection rate compared to metallic staple closure? (2) Do incisions closed with subcuticular monocryl and OCA exhibit decreased drainage? (3) Is there a cost difference between these two methods?

## Materials and methods

From July 2006 to July 2010, 215 patients with isolated acetabular fractures underwent acetabular fracture surgery at Saint Louis University Hospital, a level 1 trauma center in the United States of America. Patients eligible to participate in the study sustained an isolated acetabular fracture requiring operative fixation through a K-L approach. Fracture types to be included were posterior wall, posterior column, posterior column plus wall, transverse and transverse plus posterior wall. Additional inclusion criteria included: age 18–80 and the availability for follow-up for 1 year postoperatively. Exclusion criteria included: fracture pattern requiring a separate anterior incision or any secondary revision surgery to anatomically reduce the fracture, concurrent ipsilateral proximal femoral fracture, associated pelvic ring injury, or a Morel–Lavallée lesion. All patients who met inclusion criteria were invited to enroll in this randomized prospective clinical study comparing superficial skin closure with metallic skin staples versus skin closed using a running subcuticular monocryl suture (Ethicon, Somerville, NJ, USA), then sealed with OCA.

Patients who consented to participate in the study and met all inclusion criteria were randomized to two treatment groups. One group was randomized to superficial closure of the K-L incision with a running subcuticular 3–0 monocryl suture then sealed with OCA skin adhesive. The other group underwent standard skin closure with metallic skin staples. The treatments were randomized according to a computer based randomization program. The patient was blinded to the treatment prior to the surgery and the attending surgeon was informed of the closure method just prior to the surgical procedure.

A priori power analysis was performed, using a difference in infection rate of 5 % [20, 21], indicating that a minimum sample size of 90 patients (45 per group) was required to show a significant difference at the *p* < 0.05 level with 80 % power. To ensure the minimum of 45 patients within each group with sufficient follow-up, a total of 105 consecutive patients were enrolled (staples *n* = 53, and OCA *n* = 52). Three eligible patients solicited for the study declined to participate and did not enroll; two patients (1 staples and 1 OCA) were excluded after enrolling when it was recognized that they did not meet inclusion criteria. All of the remaining enrolled patients, a total of 52 patients in the metallic staples and 51 patients in the OCA group, were available for final analysis and were followed for 1 year. Patient demographics of the two groups were similar (Table 1) and fracture types were similarly distributed between groups (*p* = 0.84). No patients were lost to follow-up.

**Table 1:** Comparison between metallic staples and OCA groups

	Staples (*n* = 52)	OCA (*n* = 51)	*p* value
Age (years^a^)	37.3	39.9	0.257
BMI			
<25	11	17	0.165
≥25	41	34	
Sex			
Male	37	31	0.267
Female	15	20	
Side			
Right	31	30	0.935
Left	21	21	
Time to surgery (days^a^)	5.6	5.7	0.848
Time to dry wound (days^a^)	5.9	4.2	0.032
Time to dry drain holes (days^a^)	4.1	2.9	0.218
Deep drain removal (days^a^)	2.7	2.8	0.338
Superficial drain removal (days^a^)	3.0	3.3	0.251
Deep drain output (cc^a^)	174	192	0.604
Superficial drain output (cc^a^)	201	205	0.340
Incision vacuum dressing	12	7	0.204
Drain hole vacuum dressing	11	15	0.335
Infection	2	0	0.495
Hospital days^a^	14.5	13.1	0.945

*BMI* body mass index

^a^Mean values

With the exception of the skin closure method, the same treatment protocol was implemented for all patients. Unstable hips were placed in balanced skeletal traction prior to the surgical procedure. A standard preoperative anti-coagulation regimen was utilized on all patients with enoxaparin sodium 40 mg (Lovenox, Sanofi-aventis, Bridgewater, NJ, USA) injected subcutaneously once per day. The enoxaparin sodium was withheld 24 h prior to surgery. A standard K-L surgical approach, without extension or trochanteric osteotomy, was used in all cases. All surgical procedures were performed by fellowship trained orthopedic traumatologists specializing in acetabular and pelvic fracture surgery. As per hospital protocol, all study patients received preoperative antibiotics. A standard perioperative prophylactic antibiotic protocol utilizing a weight-based dose of cefazolin was administered; alternatively vancomycin was administered to patients with a drug allergy to cefazolin. Intraoperatively all patients received identical deep closure of the fascia using (0) vicryl (Ethicon, Somerville, NJ, USA) interrupted sutures and 2–0 monocryl interrupted subdermal sutures. Two suction canister 1/8 inch drains were placed: sub-fascial (deep) and extra-fascial (superficial). A standard occlusive gauze dressing was applied to all wounds. Antibiotics were continued until the suction canister drains were removed. The subcutaneously injected enoxaparin sodium 40 mg was continued 48 h postoperatively for 3 months or until the patient attained a full ambulatory status.

The operatively placed gauze dressing was removed and replaced on postoperative day 2. The dressing was inspected and reapplied every 24 h thereafter until the surgical wound was completely dry. In addition, the suction canister drains were removed when the 24-h output was less than 50 cc of fluid. A separate occlusive dressing was placed on the suction canister drain holes and changed daily until completely dry. Wounds or drain holes producing persistent copious drainage, defined as saturation of a dressing every 8 h, had a vacuum-assisted closure (KCI, San Antonio, TX, USA) device placed on either the incision or drain holes. The vacuum dressing was removed after 48 h and the wound reassessed. All wounds were assessed for infection. We defined infection as persistent purulent drainage, surrounding erythema, and positive intraoperative wound cultures. Patients were not discharged from the hospital until the incision and drain holes were completely dry. At 2 weeks, patients returned to the clinic for staple removal or wound inspection. In addition, patients were evaluated in the clinic at 6 weeks, 3 months, 6 months, and 1 year. All patients were followed clinically for 1 year.

The primary outcome measures of this study were wound infection and time to a dry surgical wound. Additional secondary outcome measures included: time to dry drain holes, deep and superficial drain outputs, vacuum-assisted dressing application to the incision and drain holes. In addition, the patients in the two groups were analyzed for the demographics of age, sex, and body mass index (BMI), as well as fracture type. Additional general data estimated included the number of staplers for skin closure in the staples group and the additional numbers of suture packs and operating room time (with a hospital patient charge of $929 per 15-min increment) for the OCA group.

Statistical analysis was performed with SPSS version 12.0 (SPSS Inc., Chicago, IL, USA). Fisher’s exact test was used to make comparisons between the two groups comparing infection rate. Pearson’s Chi-square test was utilized when comparing categorical data. The Mann–Whitney *U* test was also used to make pairwise comparisons between groups when appropriate. Statistical significance was set at *p* < 0.05. Costs were evaluated using descriptive methods.

## Results

Two deep infections (4 %) developed in the immediate postoperative period in the metallic staples group, requiring multiple irrigations and debridements with delayed closure over antibiotic beads (Table 1). Both of these patients (male, age 25 years, and female, age 46 years) were healthy, without medical co-morbidities, and had sustained isolated acetabular fractures (posterior wall and transverse plus posterior wall, respectively) in a motor vehicle accident. BMI was greater than 25 in both patients, as was the case for the vast majority of patients in this series (Table 1). No infections developed in the group closed with running subcuticular monocryl suture and OCA. However, this difference in infection rates between the two groups was not statistically significant (*p* = 0.495). There were no instances of wound dehiscence, superficial infection or late infection.

Incisions closed with running subcuticular monocryl suture and OCA were clinically dry in 4.2 days, as compared to 5.85 days for the metallic staples group (Table 1). This difference was statistically significant (*p* = 0.032). No statistical difference between the two groups was found when comparing days from surgery to dry drain holes (*p* = 0.218).

Twelve patients in the staples group and 6 patients in the OCA group had vacuum-assisted dressings placed on their surgical wounds due to persistent drainage. In addition, 11 patients in the metallic staples group and 15 patients in the OCA group required a vacuum-assisted dressing for persistent drainage from the drain holes. However, these differences for application of a vacuum-assisted dressing to the incision or drain holes were not statistically significant (*p* = 0.204 and 0.335, respectively). All wounds treated with negative pressure dressings were dry after 48 h of vacuum therapy. There was no statistical difference between the two groups (Table 1) when comparing time of deep drain retention (*p* = 0.338), time of superficial drain retention (*p* = 0.251), output of deep drain (*p* = 0.604), output of superficial drain (*p* = 0.340), and length of hospital stay (*p* = 0.945).

In general, two skin staplers were required for wound closure in the staple group at a hospital charge to the patient at our institution of $20.50 each. For the OCA group, additional suture, averaging $2.90 was required, as well as one additional operating room increment of 15 min. The result was an increased average patient charge of approximately $900 in the OCA group. However, due to the multitude of variables in the way hospital bills are generated and paid in our system, as well as the variations in patient insurance coverages, it could not be determined how—or if—this increased hospital charge translated into actual cost to the patient.

## Discussion

Metallic skin staples are currently considered the standard in superficial closure of surgical wounds in total hip arthroplasty. Recently, several studies have challenged the superiority of metallic skin staples when compared to skin closure with OCA with and without sutures [3, 11–13]. OCA has also been shown to have bacteriostatic properties, making it potentially advantageous in the use of surgical wound closures [15–17]. Surgical fixation of posterior acetabular fractures requiring a K-L approach necessitates a large dissection through traumatized soft tissue planes. The benefits of wound closure with OCA and suture demonstrated in the total hip arthroplasty literature were thought to be applicable to wound closure in fractures of the acetabulum.

Multiple studies done by Quinn et al. [15, 16] have demonstrated that OCA is not only safe in a contaminated wound model, but also provides a sealant over the wound that is bacteriostatic to many common skin and hospital bacteria. This effect was demonstrated clinically by Khurana et al. [12] in patients undergoing total hip arthroplasty, applying only OCA to the surgical incision without any other dressing. No infections were reported by Khurana et al., demonstrating the bacteriostatic sealant effect of OCA.

Kahn et al. [11] randomized patients undergoing total hip arthroplasty into three groups to receive metallic staples, 3–0 monocryl suture, or OCA and found no statistical difference in outcomes among the groups. One patient in the OCA group developed a superficial wound infection requiring debridement. In addition, Livesey et al. [13] randomized 90 patients to either OCA or metallic staple closure in total hip arthroplasty and found no difference in infection, complication rates, patient satisfaction, or cosmetic appearance. A recent meta-analysis of orthopedic patients by Smith et al. [3] demonstrated a relative risk of 3.83 when comparing the chance of developing a superficial wound infection in wounds closed with staples versus sutures. A hip surgery subgroup analysis by these authors showed an even higher relative risk of 4.79 for metallic staples versus suture closure [3]. A randomized trial by Shetty et al. [14] comparing metallic staple versus running vicryl suture closure in hip fractures did show a statistically significant difference in infection rate favoring suture closure.

These reported results indicate that closure with suture and OCA may confer a clinical advantage. Therefore, the application of topical OCA to the wound may prevent colonization of the wound that occurs postoperatively and with routine dressing changes. In addition, metallic skin staples may serve as a cutaneous foreign body that can become colonized by nosocomial bacteria in an immobilized patient. We did not find a statistical difference between the two groups. However, it is possible that a clinically important difference does exist (type II error), as no patients closed with OCA developed a deep infection compared to two patients with deep infections in the staples group.

We are unaware of any randomized controlled trial in the orthopedic literature with a larger sample size evaluating these two closure techniques. However, the still relatively small number of patients in this study is its most important limitation. Multiple recent publications have shown infection rates ranging from 5 to 7 % using the K-L approach [19–22]. Our randomized controlled clinical study was performed at a single level 1 trauma center and is sufficiently powered to show a 5 % difference in infection rate. However, the historical data of Letournel and Judet [23] showed an infection rate of 3.2 % in acetabular fracture surgery using the K-L approach. Although the exact causes of this increased infection rate are unknown to us, it may reflect the ongoing evolution of more virulent antibiotic-resistant strains of bacteria. In any case, it is possible that with more patient numbers we would have shown a statistically significant difference. Another limitation was our failure to control for potential medical co-morbidities between groups. However, overall, this was a relatively young and healthy group of fracture patients.

There was a statistically significant difference in time from surgery to dry incision favoring the OCA group. This effect was likely attributable to the sealant effect of OCA on the wound. Although there was not a statistically significant difference in vacuum-assisted dressing application to either the incision or drain holes, clinically the metallic staples group required more vacuum dressings on the incision and the OCA group required more vacuum-assisted dressings on the drain holes. Few other studies have examined wound drainage as an endpoint. We considered wound drainage to be very important, since a draining wound is always at risk for infection, and patients with a draining wound are not discharged from the hospital, thereby prolonging hospital stay. The application of negative pressure dressings to non-infected surgical wounds was highly effective in our series. All wounds treated with negative pressure dressings were dry after 48 h of vacuum therapy. This technique decreases dressing changes, potentially mitigating wound contamination and skin maceration from saturated gauze dressings. Livesey et al. [13] examined wound drainage at 3 days postoperatively and noted drainage in 39.5 % of the skin adhesive patients and 51.3 % of the staples patients in total hip arthroplasty; this difference was not statistically significant. However, Khan et al. [11] examined wound drainage accumulated on total hip arthroplasty dressings and noted a statistically significant increase in the drainage of wounds closed with metallic staples. The application of OCA to a surgical wound appears to clinically reduce the amount of drainage and may improve wound healing and decrease the number of dressing changes required.

We know of no other study that has looked at the differential in the cost of these two closure methods. One study has shown that wound closure with skin sutures takes longer than with staples, 12 versus 4.8 min on average, respectively [2]. However, the type of orthopedic surgery within or between groups was not controlled, nor were the costs described. Another study showed that closure of the skin with staples (requiring only 30 s) was significantly faster than with OCA in total hip arthroplasty patients [11]. However, their skin closure time for the OCA was much quicker (only 100 s) than in our patients, most likely because they applied OCA in two layers with a 15-s delay between applications to allow polymerization, rather than the one layer of OCA over subcuticular closure technique that we used. We found that an increased hospital charge exists in the OCA group at our institution, purely because of the added time to insert the running subcuticular suture, which resulted in an additional $929 chargeable 15-min increment. However, depending on insurance coverages and other operating room variables that affect total operating room time, often there is no real additional patient cost, hospital cost or hospital reimbursement.

In conclusion, no statistically significant difference was detected in our primary endpoint of wound infection when comparing superficial wound closure with metallic staples versus running subcuticular monocryl suture and sealed with OCA in acetabular fracture surgery. However, our results show that the running subcuticular monocryl suture and OCA closure led to a dry incision more quickly than metallic staples (*p* = 0.032), and thereby may assist in minimizing hospital stay. In addition, negative pressure dressings can be used to safely and effectively treat a non-infected draining surgical wound. Closure with OCA and subcuticular monocryl showed no clinical disadvantages and appears to have a clinical advantage when compared to standard metallic staple skin closure in acetabular fracture surgery. However, additional patient costs may be incurred.
